# Farming as food as medicine in Tanzania: reviving traditional knowledge for health, nutrition, and sustainability

**DOI:** 10.3389/fnut.2026.1905908

**Published:** 2026-08-27

**Authors:** Leonard R. Ndibalema, Joseph Kalonga

**Affiliations:** 1 Department of Agricultural Sciences, College of Agriculture, Mwalimu Nyerere University of Agriculture and Technology, Musoma, Tanzania; 2 The Department of Crop Science and Beekeeping Technology, University of Dar es Salaam, Dar es Salaam, Tanzania

**Keywords:** agroecology, food as medicine, indigenous crops, nutrition-sensitive agriculture, public health nutrition, Tanzania

## Abstract

Tanzania faces a complex nutrition and public health challenge characterized by persistent undernutrition, micronutrient deficiencies, and an increasing burden of diet-related non-communicable diseases. At the same time, traditional farming systems that historically supplied diverse, nutrient-dense foods are being weakened by urbanization, climate variability, market pressures, and dietary transitions. This narrative review examines farming as food as medicine in Tanzania by synthesizing literature on indigenous crops, agroecology, agrobiodiversity, dietary diversity, public health nutrition, and sustainable food systems. The review highlights how traditional crops such as lablab, cowpea, sorghum, millet, cassava leaves, and indigenous vegetables contribute to dietary quality, preventive health, climate resilience, and cultural continuity. It further discusses the roles of social determinants, gender, public health professionals, nutrition-sensitive agriculture, and policy integration in strengthening food-based health strategies. The review argues that smallholder farming systems should be understood not only as sources of food and income, but also as preventive public health infrastructure. Revitalizing traditional food systems and agroecological farming can contribute to improved nutrition, reduced disease risk, environmental sustainability, and more resilient livelihoods in Tanzania.

## 1 Introduction

Tanzania is a lower-middle-income country in East Africa where agriculture remains the backbone of livelihoods, employment, and rural development. Approximately two-thirds of the population depend directly or indirectly on smallholder agriculture for food, income, and household wellbeing, making agricultural systems fundamental determinants of nutrition and public health. Despite this agricultural dependence, Tanzania continues to experience a persistent triple burden of malnutrition characterized by child undernutrition, widespread micronutrient deficiencies, and a rapidly increasing prevalence of overweight, obesity, and diet-related non-communicable diseases (NCDs), including hypertension, diabetes mellitus, and cardiovascular diseases (1–3). These overlapping nutritional and health challenges threaten progress toward national development goals and the Sustainable Development Goals (SDGs), particularly SDG 2 (Zero Hunger), SDG 3 (Good Health and Well-being), and SDG 12 (Responsible Consumption and Production) (4, 5).

The persistence of these health burdens reflects profound transformations occurring across Tanzania’s food systems. Historically, Tanzanian diets were dominated by diverse, minimally processed foods derived from local farming systems, including sorghum, millet, maize, cassava, sweet potatoes, legumes, indigenous leafy vegetables, fruits, and traditional medicinal plants (6, 7, 80). These foods supplied dietary fibre, high-quality plant proteins, essential vitamins, minerals, antioxidants, and numerous bioactive phytochemicals that contributed to immune function, healthy growth, maternal nutrition, and protection against chronic diseases (8–10)). However, rapid urbanization, demographic change, globalization of food markets, climate variability, changing lifestyles, and increasing availability of highly processed foods have altered dietary patterns, resulting in declining dietary diversity and greater exposure to dietary risk factors associated with obesity and other NCDs (11–14).

Increasing recognition has therefore been given to the concept of *food as medicine*, which emphasizes the preventive and therapeutic value of healthy diets (81). Nevertheless, the foods capable of promoting health originate from agricultural systems, suggesting that farming itself should be viewed as an upstream determinant of human health. This broader perspective *farming as food as medicine* extends beyond nutrition by recognizing that agricultural practices influence the diversity, quality, accessibility, affordability, cultural acceptability, and medicinal value of foods consumed by households (12). In countries such as Tanzania, where healthcare resources remain constrained and prevention is substantially more cost-effective than treatment, strengthening agroecological farming systems and revitalizing indigenous crops (7), provide important opportunities for improving nutrition security, preventing chronic diseases, enhancing ecosystem resilience, and promoting sustainable livelihoods (15, 16).

Although considerable research has examined individual components of this relationship, the existing literature remains highly fragmented. Previous reviews have generally focused on agricultural production, nutrition-sensitive agriculture, food systems, indigenous foods, medicinal plants, public health nutrition, or sustainable diets as separate fields of inquiry. Other reviews have concentrated on specific crops, nutritional composition, food security, or disease prevention without explicitly examining the interconnected pathways linking agricultural production systems, food environments, dietary quality, human health, and environmental sustainability. Consequently, current evidence provides valuable disciplinary insights but offers limited understanding of how these domains interact as components of an integrated socio-ecological system capable of simultaneously improving nutrition, health, and sustainable development (17, 18).

This fragmentation represents an important conceptual gap because agricultural interventions rarely influence health through a single pathway. Instead, climate conditions, agricultural policies (18, 82), markets, gender relations, indigenous knowledge, biodiversity, farming practices, food processing, consumer behaviour, and healthcare systems interact dynamically to determine dietary patterns and health outcomes. Ignoring these multidimensional relationships limits the development of integrated policies capable of addressing malnutrition, diet-related diseases, environmental degradation, and declining agricultural resilience simultaneously (19, 20, 83).

**Figure 1:**
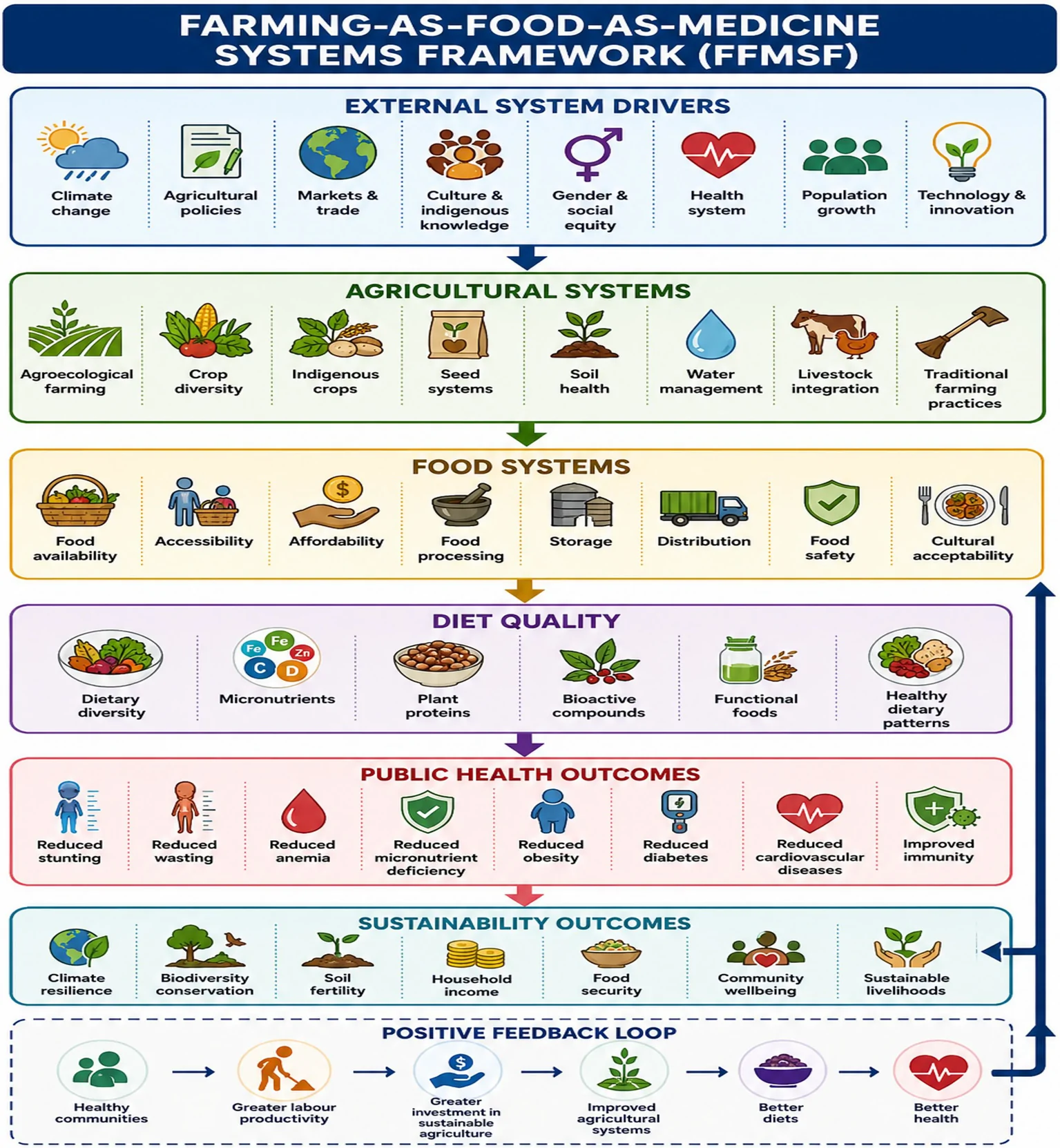
Proposed Farming-as-Food-as-Medicine Systems Framework (FFMSF) illustrating the multidirectional relationships between external system drivers, agricultural systems, food systems, diet quality, public health outcomes, and sustainability outcomes.

To address this gap, the present narrative-integrative review proposes the Farming-as-Food-as-Medicine Systems Framework (FFMSF) as a new conceptual synthesis that unifies agriculture, nutrition, public health, and sustainability within a single systems perspective. Unlike previous reviews that predominantly describe linear relationships between agriculture and nutrition, the FFMSF conceptualizes farming as a complex adaptive system characterized by multiple interacting drivers, mediating pathways, feedback mechanisms, and sustainability outcomes (Figure 1). The framework illustrates how climate, governance, markets, socio-cultural factors, and health systems shape agricultural production; how agricultural systems influence food systems and diet quality; and how improved health and community wellbeing subsequently reinforce resilient agricultural development through positive feedback loops (21, 84).

Guided by this framework, this review synthesizes current evidence on the role of farming as food as medicine in Tanzania by integrating findings across agriculture, nutrition, food systems, public health, and environmental sustainability. Specifically, the review aims to (i) examine how agricultural systems influence dietary quality and nutritional outcomes; (ii) evaluate the contribution of indigenous crops and agroecological practices to disease prevention and public health; (iii) analyse the interactions between agriculture, food systems, environmental sustainability, and community wellbeing; and (iv) develop a unified conceptual framework capable of informing future interdisciplinary research, policy development, and integrated agricultural and health interventions in Tanzania and comparable low and middle-income countries. Existing literature can be categorized into four major streams. First, agricultural studies have focused primarily on crop productivity, food security, and farm income. Second, nutrition research has largely examined dietary quality and micronutrient deficiencies without explicitly considering agricultural production systems. Third, food systems research has investigated food environments and market transformation but has seldom incorporated ecological processes or preventive healthcare. Finally, public health research has predominantly emphasized dietary behaviour and clinical outcomes while paying limited attention to farming systems as upstream determinants of health. Consequently, no previous review has synthesized these domains within a unified systems framework incorporating mediating pathways, moderating factors, feedback mechanisms, and sustainability outcomes. This conceptual gap provides the basis for developing the Farming-as-Food-as-Medicine Systems Framework (FFMSF).

### 1.1 The farming-as-food-as-medicine systems framework (FFMSF)

The Farming-as-Food-as-Medicine Systems Framework (FFMSF) was developed to address a major conceptual limitation within the existing literature. Previous reviews have generally examined agriculture, nutrition, food systems, public health, and environmental sustainability independently or have assumed simple linear relationships between agricultural production and nutritional outcomes. Such approaches inadequately capture the complexity of food systems, where multiple social, ecological, economic, cultural, and institutional factors interact simultaneously to influence human health. The FFMSF conceptualizes farming as a complex adaptive socio-ecological system in which agricultural production constitutes the foundation of population health. External drivers including climate change, agricultural policies, market dynamics, indigenous knowledge, gender relations, technological innovation, and health systems—shape farming practices and determine the diversity, resilience, and sustainability of agricultural production systems. These agricultural systems subsequently influence food-system performance by determining food availability, accessibility, affordability, quality, processing, storage, and cultural acceptability.

Food-system characteristics directly affect dietary quality through their influence on dietary diversity, micronutrient intake, plant-based proteins, functional foods, and bioactive compounds. Improved diet quality contributes to better nutritional status and reduced risks of child undernutrition, micronutrient deficiencies, obesity, diabetes, cardiovascular diseases, and other diet-related non-communicable diseases. Beyond these health benefits, healthier diets support broader sustainability outcomes by strengthening food security, household incomes, biodiversity conservation, soil health, climate resilience, and community wellbeing. A distinguishing feature of the FFMSF is the incorporation of a reinforcing feedback mechanism. Improved health enhances labour productivity, educational attainment, and household economic capacity, enabling greater investment in sustainable farming practices, agricultural innovation, and environmental stewardship. These investments further improve agricultural system performance, thereby strengthening food systems and generating a continuous cycle linking agriculture, nutrition, public health, and sustainability.

By integrating these multidimensional pathways into a single conceptual model, the FFMSF advances existing theory beyond descriptive agriculture–nutrition linkages. It provides a holistic systems framework that can guide interdisciplinary research, inform integrated agricultural and health policies, and support the design of interventions that simultaneously improve nutrition, disease prevention, environmental sustainability, and resilient food systems. The framework therefore constitutes the principal theoretical contribution of this review.

## 2 Review methodology

### 2.1 Review design

This review employed an integrative narrative review methodology to critically examine the emerging concept of *farming as food as medicine* and to synthesize evidence linking agricultural systems, food systems, nutrition, public health, and sustainability. An integrative review was selected because the subject spans multiple disciplines and requires the integration of empirical research, systematic reviews, conceptual literature, and policy evidence to generate new theoretical understanding rather than merely summarize previous findings (22–24). Unlike systematic reviews, which typically address narrowly defined questions through quantitative evidence synthesis, integrative reviews accommodate heterogeneous evidence and facilitate conceptual innovation by identifying relationships, inconsistencies, and emerging patterns across diverse research traditions (25, 26).

Accordingly, the objective of this review extended beyond describing existing knowledge to developing a systems-based conceptual framework that explains how agricultural systems influence dietary quality, public health, and sustainability through interconnected biological, social, environmental, and policy pathways. The review therefore adopted a thematic and interpretive synthesis approach that emphasized explanation, comparison, and theory development rather than simple aggregation of published findings.

### 2.2 Literature search and study selection

A comprehensive literature search was conducted to identify multidisciplinary evidence relating to agriculture, food systems, nutrition, public health, and sustainability. Searches were undertaken across six internationally (27), recognized bibliographic databases Scopus, Web of Science, PubMed, CAB Abstracts, AGRIS, and Google Scholar which collectively provide extensive coverage of agricultural sciences, nutrition, medicine, environmental sciences, and social sciences (28). Because important evidence in food systems research is frequently disseminated through technical reports and institutional publications, the search was complemented with grey literature (29), from FAO, WHO, UNICEF, IFAD, WFP, HLPE, IPES-Food, the World Bank, and relevant Tanzanian policy documents (30).

Searches combined controlled vocabulary and free-text keywords using Boolean operators (31). Major search terms included *food as medicine*, *nutrition-sensitive agriculture*, *agroecology*, *traditional food systems*, *indigenous crops*, *dietary diversity*, *agrobiodiversity*, *smallholder farming*, *sustainable diets*, *non-communicable diseases*, *Tanzania*, and *sub-Saharan Africa*. No publication-year restrictions were applied because the review sought to integrate both seminal literature that established theoretical foundations and recent studies reflecting contemporary developments in sustainable food systems.

The search process identified 487 publications. Following removal of 82 duplicate records, 405 unique publications were screened by title and abstract. During initial screening, 268 publications were excluded because they were unrelated to the review objectives, focused exclusively on clinical interventions without agricultural relevance, lacked nutrition or public health outcomes, or consisted of editorials, conference abstracts, commentaries, or duplicate publications. The remaining 137 full-text articles underwent detailed eligibility assessment, resulting in exclusion of 48 studies that lacked sufficient methodological quality, did not adequately examine agriculture–nutrition–health relationships, or presented findings with limited conceptual relevance. Consequently, 89 publications were included in the final synthesis.

**Figure 2:**
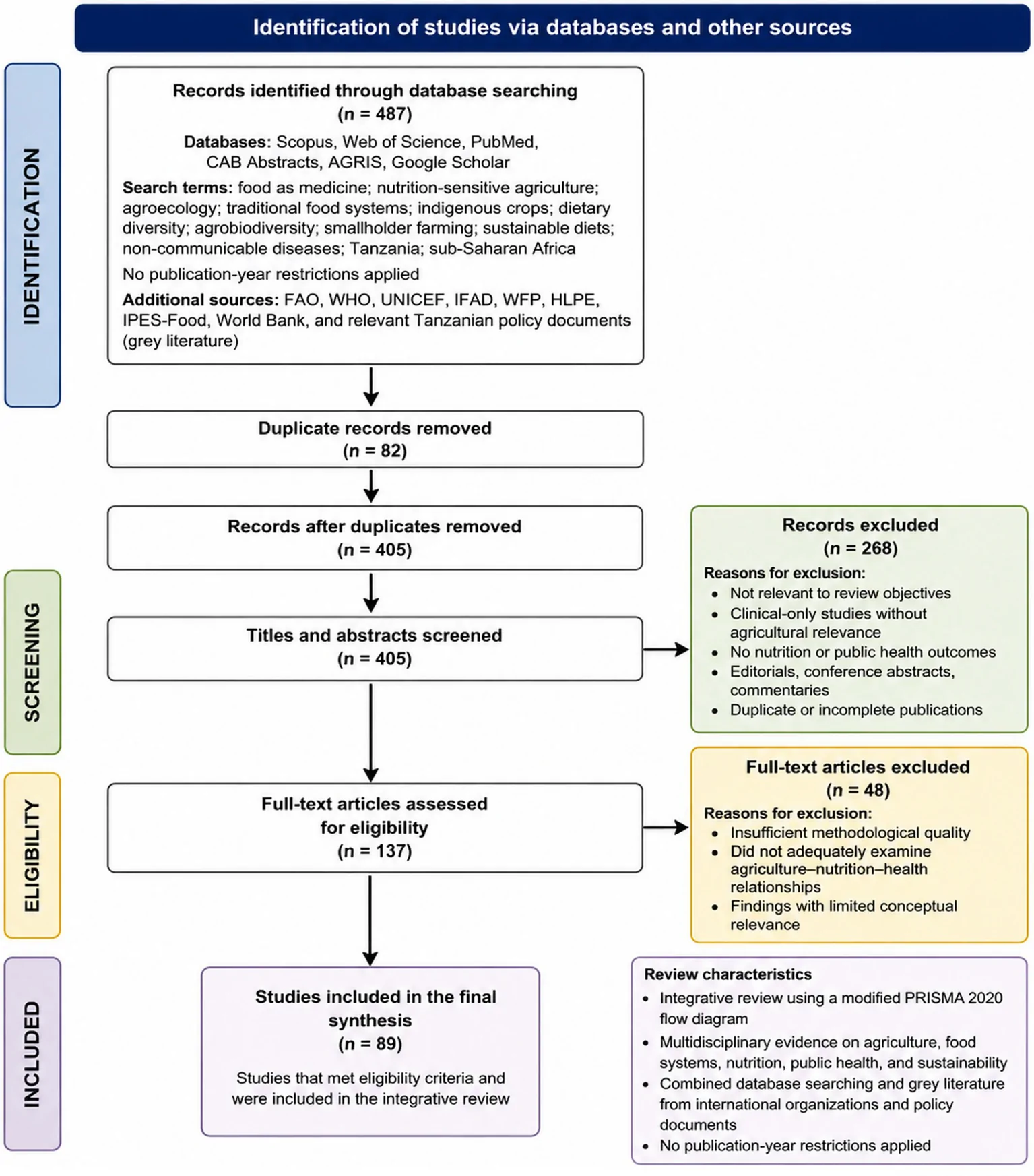
PRISMA flow diagram illustrating the literature search and study selection process used in this integrative review.

Although this review followed an integrative rather than systematic methodology, the literature selection process is presented using a modified PRISMA flow diagram (32) (Figure 2) to improve methodological transparency and reproducibility, consistent with recommendations for high-quality integrative reviews (24, 26).

### 2.3 Eligibility criteria

Eligibility criteria were established before the literature search to ensure consistency and minimize selection bias (33). Publications were included when they examined relationships among agricultural production, food systems, nutrition, public health, or sustainability and contributed empirical, theoretical, or policy evidence relevant to the objectives of the review. Eligible studies included investigations of agroecological farming, indigenous crops, dietary diversity, nutrition-sensitive agriculture, food environments, and sustainable food systems conducted in Tanzania, sub-Saharan Africa, or other comparable low and middle-income settings. Systematic reviews, meta-analyses, observational studies, qualitative investigations, mixed-methods research, and internationally recognized policy reports were all considered eligible because of the interdisciplinary nature of the review.

Studies were excluded when they focused exclusively on pharmaceutical or clinical interventions without consideration of food systems, investigated agricultural productivity without nutrition or health outcomes, consisted solely of conference abstracts, editorials, commentaries, or opinion papers, lacked adequate methodological information, or duplicated previously published findings. These criteria ensured that all included evidence contributed directly to understanding the multidimensional relationships among agriculture, nutrition, health, and sustainability.

### 2.4 Quality assessment

Methodological quality was evaluated using an evidence hierarchy rather than formal numerical scoring, reflecting established guidance for integrative reviews (23, 24). This approach recognizes that evidence derived from different study designs contributes differently to conceptual development while allowing incorporation of policy documents and interdisciplinary research that would ordinarily be excluded from conventional systematic reviews (34).

Included studies were therefore interpreted according to methodological rigor, consistency of findings, contextual relevance, and theoretical contribution. Systematic reviews, meta-analyses, randomized controlled trials, and longitudinal cohort studies were regarded as providing the strongest evidence. Cross-sectional, observational, qualitative, and mixed-methods studies were considered to provide moderate evidence, particularly for understanding contextual and behavioural dimensions. International policy reports, expert consensus documents, and conceptual papers were used primarily to support theoretical interpretation and policy implications.

Importantly, evidence was not weighted equally. Greater interpretive emphasis was placed on higher-quality empirical studies when drawing conclusions regarding agriculture–nutrition–health relationships, whereas conceptual papers and policy reports were used primarily to explain mechanisms, identify implementation challenges, and contextualize empirical findings. This evidence-weighting approach strengthened the credibility of the conceptual synthesis while acknowledging the heterogeneous nature of the available literature.

### 2.5 Data synthesis

Data synthesis followed an interpretive thematic approach designed to generate new conceptual understanding rather than merely summarize previous research (24, 26). Extracted evidence was organized into five interconnected domains comprising agricultural systems, food systems, diet quality and nutrition, public health outcomes, and sustainability outcomes. Within each domain, studies were systematically compared to identify areas of convergence, conflicting findings, contextual variation, and potential causal mechanisms across diverse agroecological and socioeconomic settings.

Rather than presenting evidence sequentially on a study-by-study basis, synthesis emphasized comparison, critical evaluation, and interpretation. Particular attention was given to explaining why findings differed across geographical regions, production systems, market environments, and population groups, and to identifying the contextual factors that strengthened or weakened agriculture–nutrition pathways. Climate variability, market integration, gender dynamics, indigenous knowledge, institutional support, and policy environments were consistently examined as interacting determinants influencing both dietary quality and health outcomes (35).

Finally, evidence from all thematic domains was integrated to develop the proposed Farming-as-Food-as-Medicine Systems Framework (FFMSF). The framework extends conventional linear agriculture-food-health models by incorporating multidirectional causal pathways, mediating and moderating factors, reinforcing feedback mechanisms, and sustainability outcomes. In doing so, it provides a novel systems-based conceptual model for understanding how agricultural biodiversity, food systems, and public health interact within complex socioecological environments (36).

## 3 Food as medicine: clinical and public health perspectives

The concept of food as medicine has evolved from a nutrition-centred approach into a broader public health strategy that recognizes food systems as fundamental determinants of health, disease prevention, and healthcare sustainability. Diets rich in whole grains, legumes, fruits, vegetables, nuts, and seeds provide essential macro and micronutrients (19) together with dietary fibre, phytochemicals, and other bioactive compounds that support immune function, metabolic regulation, gut microbiome diversity, and reduced risk of both infectious and chronic diseases (11, 14, 37). Consequently, nutrition interventions are increasingly shifting from treatment-oriented approaches toward preventive food-based strategies that position agriculture and food systems as upstream determinants of population health (38–43).

Evidence consistently demonstrates that healthy dietary patterns reduce the burden of micronutrient deficiencies, child undernutrition (44, 85), obesity, hypertension (45), type 2 diabetes, and cardiovascular disease (3, 45, 46). However, direct evidence linking specific agricultural production systems or indigenous crops to measurable clinical outcomes remains comparatively limited. Most studies focus on dietary diversity, household food security, and nutrient intake rather than objective biomarkers such as haemoglobin concentration, serum ferritin, glycated haemoglobin (HbA1c), lipid profiles, inflammatory markers, or gut microbiome composition. Thus, confidence is high regarding the benefits of diversified diets for nutritional quality but remains moderate regarding their direct long-term clinical effects, highlighting an important research gap.

Recent evidence further demonstrates that food-based interventions such as medically tailored meals, produce prescription programmes, and nutrition-sensitive community interventions can significantly improve dietary quality, glycaemic control, blood pressure, and healthcare utilization among vulnerable populations. Although most evidence originates from high-income countries, these findings reinforce the biological plausibility that diversified agricultural production can contribute to disease prevention through improved dietary quality. Future research should evaluate similar interventions using indigenous foods within African food systems (39, 40).

Nevertheless, important evidence gaps remain. Most available studies rely on cross-sectional dietary assessments rather than longitudinal intervention trials capable of demonstrating causal relationships between agricultural diversification, food consumption, biomarkers of nutritional status, and long-term health outcomes. Consequently, stronger interdisciplinary research integrating agriculture, nutrition, epidemiology, and public health is required to establish robust evidence supporting farming as food as medicine (38, 42).

In Tanzania, the food-as-medicine concept is deeply rooted in indigenous farming systems that integrate nutrition and health. Traditional foods-including lablab (*Lablab purpureus*), cowpea (*Vigna unguiculata*), sorghum, millet, cassava leaves, amaranth, and other indigenous vegetables provide high-quality plant protein, dietary fibre, iron, zinc, folate, calcium, and numerous phytochemicals associated with improved nutritional status and reduced risk of diet-related diseases (9, 10, 86, 87). Nevertheless, rapid urbanization, market globalization, and increased consumption of refined cereals, sugar-sweetened beverages, processed foods, and unhealthy fats have accelerated dietary transition, contributing to the growing burden of obesity and non-communicable diseases (3, 11, 47). Collectively, the evidence supports an integrated systems perspective in which diversified agriculture strengthens food environments, improves dietary quality, and promotes preventive healthcare and sustainable development, forming the conceptual foundation of the proposed Farming-as-Food-as-Medicine Systems Framework (FFMSF) (6, 48–50).

## 4 Agroecology, agrobiodiversity, and sustainable diets

Agroecology provides a strong foundation for the Farming-as-Food-as-Medicine Systems Framework (FFMSF) because it integrates agricultural production, ecological sustainability, dietary quality, and public health within a single systems perspective. Rather than viewing agriculture solely as a means of maximizing yields, agroecology considers farms as socioecological systems that simultaneously produce food, conserve biodiversity (51), enhance soil fertility (52), strengthen climate resilience, sustain livelihoods, and improve human health. Agroecological practices including crop diversification, intercropping, agroforestry, crop rotation, crop-livestock integration, biological pest management, and soil organic matter conservation have consistently been associated with improved food security, ecosystem resilience, and dietary diversity when implemented as part of broader food-system transformation (5, 9, 53–55, 86). These findings reinforce the view that agriculture functions as an upstream determinant of nutrition, health, and environmental sustainability rather than as an isolated production sector (20, 53).

Agrobiodiversity is a principal mechanism through which agroecology contributes to sustainable diets. Diverse farming systems generally produce a wider range of cereals, legumes, fruits, vegetables, roots, tubers, and animal-source foods, thereby improving dietary diversity, which is widely recognized as a proxy for micronutrient adequacy and overall diet quality [(56); Walter (14)]. In Tanzania, indigenous and underutilized crops including lablab (*Lablab purpureus*), cowpea (86) (*Vigna unguiculata*), pigeon pea (*Cajanus cajan*), sorghum (*Sorghum bicolor*), pearl millet (*Pennisetum glaucum*), cassava leaves (*Manihot esculenta*), amaranth (*Amaranthus* spp.), and other indigenous vegetables provide complementary plant proteins, dietary fibre, iron, zinc, calcium, folate, antioxidants, and other bioactive compounds that support healthy diets while reducing dependence on a limited number of staple crops (9, 10). Thus, agrobiodiversity should be understood not only as ecological diversity but also as nutritional biodiversity, linking ecosystem integrity with human nutrition (57).

However, the relationship between agricultural diversity and nutritional outcomes is neither linear nor universal. While diversified farming generally improves household dietary diversity particularly in subsistence-oriented rural communities its nutritional benefits are influenced by income, market access, gender relations, nutrition knowledge, cultural preferences, food safety, and intra-household food allocation. In peri-urban and market-oriented settings, dietary choices are increasingly shaped by food environments and purchasing power rather than farm production alone. Consequently, agriculture-based nutrition interventions are most effective when integrated with nutrition education, women’s empowerment, equitable market access, and supportive public health policies (53, 58). These findings indicate that food environments mediate, and social conditions moderate, the agriculture–nutrition relationship.

Climate change further reinforces the importance of agroecology for sustainable diets. Increasing climate variability threatens food production through declining crop yields, soil degradation, pest outbreaks, altered nutrient composition of crops, and food-price instability, thereby increasing the risk of malnutrition (59); (60). Diversified agroecological systems enhance resilience by improving soil organic matter, conserving moisture, increasing biological nitrogen fixation, and promoting drought-tolerant species such as lablab, cowpea, sorghum, and millet. These ecological functions directly contribute to food security and dietary resilience under changing climatic conditions. Nevertheless, climate-smart agriculture alone does not guarantee improved nutrition unless interventions explicitly address dietary quality, affordability, and equity.

Overall, current evidence strongly supports agroecology and agrobiodiversity as complementary pathways for operationalizing farming as food as medicine. Although evidence linking diversified farming to dietary diversity and household food security is robust, fewer longitudinal and intervention studies have demonstrated direct effects on clinical biomarkers such as haemoglobin, serum ferritin, glycated haemoglobin (HbA1c), inflammatory markers, lipid profiles, or gut microbiome composition. Future research should therefore strengthen causal evidence connecting agricultural biodiversity, biological mechanisms, nutrition, and long-term health outcomes. Collectively, the available literature supports the Farming-as-Food-as-Medicine Systems Framework (FFMSF) by demonstrating that agriculture, nutrition, public health, and environmental sustainability are interconnected through multidirectional and context-dependent pathways, with broad relevance for Tanzania and other low and middle-income countries undergoing dietary transition, climate change, and declining agrobiodiversity. However, the strength of these associations varies substantially across contexts. Studies conducted in subsistence-oriented farming systems generally report stronger relationships between production diversity and dietary diversity than studies in commercialized agricultural systems. This suggests that market integration acts as an important moderator within the FFMSF by weakening direct farm-to-diet pathways while strengthening market-mediated pathways (17, 61–63).

## 5 Social determinants of nutrition and health

The relationship between farming systems and health is shaped not only by agricultural production but also by a complex interplay of social, economic, institutional, and environmental determinants that influence food access, dietary behaviour, and nutritional outcomes (86). Contemporary food systems research recognizes that increasing agricultural productivity alone is insufficient to improve nutrition unless households possess the resources, knowledge, and opportunities required to access and utilize nutritious foods (3, 4, 86). Accordingly, the Farming-as-Food-as-Medicine Systems Framework (FFMSF) conceptualizes agriculture as interacting with poverty, gender, education, markets, healthcare, governance, and culture to determine population health.

Among these determinants, women’s empowerment consistently emerges as one of the strongest predictors of improved household nutrition. Women play central roles in seed selection, crop diversification, home gardening, food preparation, infant and young child feeding, and household dietary decisions. Greater decision-making power, access to productive resources, education, and agricultural extension services are associated with higher dietary diversity, improved maternal nutrition, and better child growth outcomes (4, 64). However, persistent inequalities in land ownership, financial resources, technology access, and participation in decision-making continue to limit the nutritional benefits of agricultural interventions in many low-income settings, highlighting gender equity as a critical pathway linking agriculture and health (65).

Income, poverty, and food environments further influence dietary quality. Although diversified farming can increase the availability of nutrient-rich foods, low household income often restricts access to complementary foods, healthcare, sanitation, and agricultural inputs. Poor households frequently rely on inexpensive calorie-dense staples while consuming fewer fruits, vegetables, legumes, and animal-source foods (4). Moreover, rural and urban populations experience different nutrition challenges. Rural households often benefit from their own agricultural production but remain vulnerable to seasonal food shortages and climate shocks, whereas urban populations increasingly depend on commercial food markets where processed foods, refined carbohydrates, and sugar-sweetened beverages are readily available, contributing to rising rates of overweight, obesity, hypertension, type 2 diabetes, and cardiovascular disease (3, 11).

Market access, infrastructure, and nutrition knowledge are equally important determinants. Poor rural roads, inadequate storage facilities, post-harvest losses, and weak value chains reduce the availability and affordability of fresh and nutrient-dense foods, particularly in remote communities. Conversely, efficient markets, strengthened indigenous crop value chains, and better connections between smallholders and schools or local markets can improve both livelihoods and dietary quality (High Level Panel of Experts on Food Security and Nutrition, 2019). Likewise, households with greater nutrition literacy are better able to translate agricultural production into improved diets, while nutrition-sensitive agricultural extension programmes outperform production-oriented approaches by integrating nutrition education with crop management.

Overall, the evidence demonstrates that farming-as-food-as-medicine cannot be achieved through agricultural production alone. Rather, nutrition and health emerge from interactions among farming systems, gender equity, household income, education, market environments, and public institutions. These social determinants operate as key moderators within the Farming-as-Food-as-Medicine Systems Framework (FFMSF), influencing how agricultural diversification translates into healthier diets and improved public health. Integrating women’s empowerment, nutrition education, equitable market development, social protection, and diversified agriculture is therefore essential for achieving sustainable food systems and better health outcomes, particularly in Tanzania and other low and middle-income countries undergoing rapid dietary and agricultural transformation (66).

## 6 Public health professionals, interventions, and policy pathways

Public health professionals play a pivotal role in translating the Farming-as-Food-as-Medicine Systems Framework (FFMSF) into practical interventions that improve dietary quality, prevent disease, and strengthen food-system resilience. Increasing evidence indicates that nutrition and health outcomes are influenced not only by individual food choices but also by agricultural production, food environments, education, healthcare systems, market access, and policy coordination (3, 58). Consequently, effective public health action must extend beyond nutrition counselling to address the structural factors that determine whether households can produce, access, prepare, and consume healthy foods.

This multisectoral approach requires collaboration among nutritionists, dietitians, agricultural extension officers, community health workers, environmental health practitioners, and educators. Nutritionists and dietitians provide culturally appropriate dietary guidance using locally available foods, while agricultural extension officers promote crop diversification, legume integration, soil fertility management, home gardens, post-harvest handling, and conservation of indigenous crops. Community health workers strengthen maternal and child nutrition, dietary diversity, infant feeding, and food preparation practices, whereas environmental health practitioners contribute through food safety, water quality, sanitation, and safe food storage, recognizing that nutritional outcomes depend on both adequate diets and healthy living environments (3, 66–68).

Nutrition-sensitive agriculture provides one of the most practical pathways for operationalizing farming as food as medicine. Unlike conventional production-oriented agriculture, nutrition-sensitive approaches intentionally integrate agricultural production with nutrition objectives through diversified cropping systems, home and school gardens, small livestock integration, community seed systems, nutrition education, and women’s empowerment (58). In Tanzania, prioritizing indigenous legumes, traditional cereals, and leafy vegetables can improve dietary diversity while simultaneously enhancing climate resilience and supporting sustainable livelihoods. Likewise, home-grown school feeding programmes that procure food from local smallholder farmers create stable markets while improving children’s dietary quality and educational outcomes (15).

Achieving these outcomes requires strong policy coherence across agriculture, health, education, environment, and social protection sectors. Policies that strengthen agroecological extension, indigenous seed systems, women’s access to productive resources, food safety, local procurement, and smallholder value chains can substantially enhance the contribution of agriculture to nutrition and disease prevention [High Level Panel of Experts on Food Security and Nutrition, 2019; (69)]. Within the FFMSF, public health professionals therefore function as critical connectors linking agricultural systems, food environments, dietary behaviour, and health outcomes. This integrated approach provides a practical pathway for strengthening preventive healthcare and sustainable food systems in Tanzania and other low and middle-income countries experiencing rapid dietary transition, climate change, and an increasing burden of malnutrition and diet-related non-communicable diseases.

## 7 Ethical and policy considerations

Food-as-medicine approaches must be implemented ethically. First, they should promote equity by ensuring that poor households, women, youth, and marginalized farmers have access to land, seeds, credit, extension services, and markets (72). Second, they should respect indigenous knowledge and cultural food practices rather than replacing them with externally driven dietary models. Third, they should protect agrobiodiversity by supporting local seed systems and traditional crops that are nutritionally, culturally, and ecologically valuable (9, 10). There are also important trade-offs. For example, policies that prioritize export crops or commercial monocultures may generate income but reduce land and labour available for diverse food crops. Similarly, promotion of high-yielding staples may improve calorie availability while weakening dietary diversity. Ethical decision-making should therefore consider nutrition, health equity, environmental sustainability, cultural identity, and farmer livelihoods together, rather than focusing only on yield or income (16, 69). Studies should examine seed access, cooking knowledge, youth preferences, market demand, women’s decision-making power, and institutional support. Such evidence would help policymakers and practitioners scale farming-as-food-as-medicine approaches in ways that are culturally acceptable, economically viable, and environmentally sustainable (70).

## 8 Future directions and research gaps

While the concept of farming as food as medicine has gained attention globally, there remains a significant gap in empirical evidence linking agricultural practices with measurable health outcomes in Tanzania and broader sub-Saharan Africa. Addressing these gaps is essential for guiding policy, scaling interventions, and ensuring that nutrition-sensitive farming systems achieve their full potential in improving population health.

### 8.1 Need for longitudinal and impact studies

Most studies in Tanzania and East Africa focus on cross-sectional assessments of dietary diversity, crop production, or household food security (9, 16). While informative, these studies do not capture long-term effects of diversified farming on health outcomes such as stunting, anemia, or diet-related NCDs. Future research should prioritize longitudinal and cohort studies that track households over multiple seasons or years. For example, monitoring the health outcomes of families adopting intercropping systems with indigenous legumes such as lablab and cowpea alongside cereals could provide robust evidence on how agricultural diversification influences micronutrient intake, growth, and NCD risk over time (71, 72, 78).

### 8.2 Linking specific crops to health outcomes

Despite anecdotal and historical evidence supporting the nutritional benefits of indigenous crops, there is limited quantifiable data on their impact on human health in Tanzanian contexts (73). Research should focus on first, nutrient profiling of local crops, including amino acid composition, iron, zinc, vitamin A, and bioavailability, second dietary intervention studies using local foods to assess effects on biomarkers such as haemoglobin, vitamin A status, and blood glucose regulation, third functional outcomes, such as improved cognitive development in children or reduced incidence of anemia and chronic diseases in adults. Such studies would provide scientific validation for the food-as-medicine paradigm, guiding both public health nutrition interventions and agricultural policy.

### 8.3 Evaluating cost-effectiveness and scalability

Sustainable interventions must also be economically viable and scalable (74). Current programs often lack rigorous analysis of cost-effectiveness, including comparisons between agroecology-based interventions and conventional nutrition programs or supplementation strategies. Research should examine, production costs, labour requirements, and yield stability of diversified cropping systems. Health benefits per unit cost, including reductions in stunting, micronutrient deficiencies, and NCD risk. Potential for scaling interventions through government programs, NGOs, and private sector partnerships. Such analyses are critical to inform national policy and investment decisions, ensuring that resources are directed toward interventions that are both effective and sustainable.

### 8.4 Integrating climate resilience

Tanzania is highly vulnerable to climate variability, which affects crop yields, food availability, and nutrition outcomes. Future research should explore the intersection of climate resilience, nutrition, and health, such as: How drought-tolerant legumes such as lablab and cowpea buffer against seasonal food insecurity and maintain micronutrient intake (75). Adaptive cropping systems that reduce vulnerability to climate shocks while supporting preventive health outcomes. Predictive modeling of climate impacts on nutrition-sensitive agriculture and associated health risks (16, 59). Integrating climate resilience ensures that food-as-medicine approaches remain robust in the face of environmental uncertainty.

### 8.5 Social and behavioural dimensions

Understanding behavioural and social determinants of adoption is crucial. Research should explore: Household decision-making regarding crop selection, dietary practices, and resource allocation. Gender dynamics, particularly the role of women in seed selection, home gardens, and nutrition management. Cultural preferences and barriers to adopting nutrient-dense indigenous foods (9, 64, 76). Insights from these studies can inform community-tailored interventions that maximize adoption and health impact.

### 8.6 Policy and systems research

**Figure 3:**
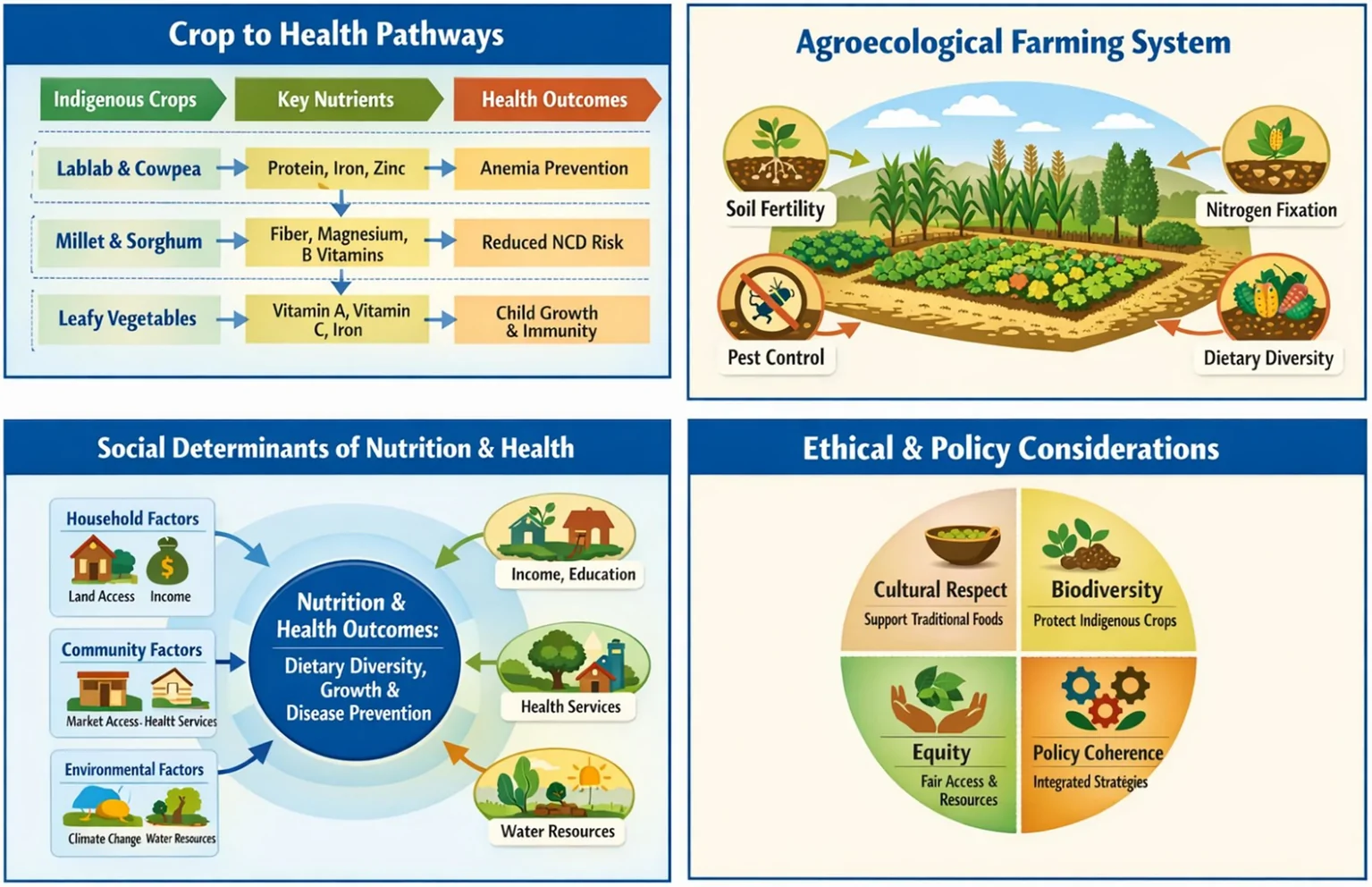
Crop-to-health pathways, agroecological farming system, social determinants of nutrition and health, and ethical and policy considerations in Tanzanian smallholder farming systems.

Finally, research should examine policy and institutional frameworks supporting the integration of agriculture and health (77). Key questions include: How do existing agricultural policies affect smallholder access to nutrient-dense crops? What mechanisms enable cross-sectoral coordination between agriculture, health, and education? How can social protection programs enhance access to locally produced, nutrient-rich foods? Answering these questions will provide evidence-based guidance for scaling food-as-medicine approaches and strengthening national food and nutrition systems. Future research in Tanzania and sub-Saharan Africa should focus on longitudinal impact studies, crop-health linkages, cost-effectiveness, climate resilience, behavioural adoption, and policy integration. Generating robust empirical evidence will strengthen the scientific foundation for farming as food as medicine, enabling interventions that are nutritionally effective, culturally acceptable, economically viable, and environmentally sustainable. Such research will empower policymakers, public health professionals, and smallholder farmers to maximize the preventive health potential of agricultural systems (Figure 3).

## 9 Conclusion

Farming as food as medicine offers a powerful framework for rethinking the role of agriculture in Tanzania’s public health and sustainability agenda (78, 79). Traditional farming systems, indigenous crops, and agroecological practices can support dietary diversity, micronutrient adequacy, disease prevention, climate resilience, and cultural continuity. However, these benefits will only be fully realized if social inequalities, weak markets, policy fragmentation, and declining traditional food knowledge are addressed. Strengthening nutrition-sensitive agriculture, indigenous crop value chains, public health education, and cross-sectoral policy coordination can position smallholder farming as preventive health infrastructure. For Tanzania, revitalizing traditional food systems is therefore not only a cultural or agricultural priority, but also a public health strategy for building healthier, more resilient, and more sustainable communities.
